# Representativeness of a German AI-enabled data network for secondary epidemiological analysis based on electronic health records

**DOI:** 10.1371/journal.pone.0339647

**Published:** 2026-01-06

**Authors:** Sabine Eichhorn, Franz Niklas Mitze, Fritz Wagner, Inga Marte Charlott Seuthe, Ralph Brinks, André Zimmermann, Mehdi Dastur, Josef Ladenbauer, Jonas Jae-Hyun Park

**Affiliations:** 1 University of Witten/Herdecke, Department of Otorhinolaryngology and Head and Neck Surgery, St. Josefs-Hospital, Hagen, Germany; 2 University of Witten/Herdecke, Medical Biometry and Epidemiology, Witten, Germany; 3 Tiplu GmbH, Hamburg/Berlin, Germany; De Montfort University Faculty of Health and Life Sciences, UNITED KINGDOM OF GREAT BRITAIN AND NORTHERN IRELAND

## Abstract

**Introduction:**

The ongoing digitalization of medicine, increased computing power and low-cost storage capacities enable the use of AI-based algorithms for epidemiological big data analysis of electronic patient records. The aim of this study was to evaluate the representativeness of a data network with infrastructure for federated machine learning (ML) across numerous German hospitals. This was done by comparing basic data from the ML data network with publicly available data from the Federal Statistical Office (DESTATIS) to test the scientific validity for future epidemiological analyses.

**Methods:**

In a retrospective epidemiological secondary analysis, 8,106,105 case files from the ML network were examined and compared to DESTATIS data regarding age, gender, length of hospital stay, ICD-10 diagnoses, and OPS codes. In addition, ICD-10 codes for substance abuse and the regional distribution were compared to examine socioeconomic confounders.

**Results:**

The variables age, gender and length of stay, as well as the most common general ICD-10 and OPS codes and ENT-specific OPS codes, showed a high level of concordance based on clinical relevance. For the ENT-specific ICD-10 codes, 2 out of 11 of the most frequent codes showed a maximum deviation of 3.71%. The analysis of socioeconomic factors and regional distribution showed no evidence for deviations.

**Discussion:**

The high level of agreement for the variables examined indicates the representativeness of the ML dataset in comparison to the DESTATIS data. This finding paves the way for future epidemiological studies based on big data, which were previously unavailable in research.

## Introduction

### Background

Randomized controlled trials, based on well-defined principles of good clinical practice, are the most reliable way of generating evidence in medical science. However, their feasibility is limited. Another crucial point is the discrepancy between patient care in well-controlled trials and the ‘real world’ quality of care [1]. These limitations necessitate the use of alternative scientific methods based on other data sources [2].

Non-interventional studies are therefore an essential component of clinical research. However, there is still much work to be done to establish generally accepted basic requirements in this field of science, although efforts are being made to improve [3]. Analyzed data can be collected either prospectively or retrospectively, and there are descriptive and analytical approaches.

Regarding well-defined data sets, prospective registries, such as cancer registries, are designed to document the existent actual standard of care. In Germany, government organizations are increasingly recognizing and promoting their importance [4]. Another source of prospectively collected data are national statistical authorities or health care providers, who often make anonymized, prospectively collected medical data available for scientific or statistical analysis. Since the introduction of the Diagnosis Related Groups (DRG) for billing purposes in Germany, a large amount of digital coding data has been documented in a register-like manner and is publicly available via the Federal Statistical Office’s GENESIS online database and the Federal Health Reporting System (GBE-Bund).

The process of creating these prospective data collections is time-consuming, and the content is limited.

As a result, other existing data, such as health records (HR), are typically used retrospectively for secondary data analysis. These types of data sets are more heterogeneous, complex, and largely based on semantic information. As a result, there is a degree of uncertainty in the analysis of such data and the evaluation of large amounts of data is limited. This generally increases the risk of questionable research practices [5]. In response to this problem, publication standards were developed (STROBE), for example for epidemiological issues years ago and have been refined continuously [6].

The increasing digitalization of medicine, improvements in computing power, and inexpensive, large-scale storage now enable epidemiologic analysis of large quantities of electronic health records (EHRs) [7]. In addition, the combination of digital ‘big data’ and algorithms based on artificial intelligence (AI) promotes new approaches to reach novel scientific conclusions [8]. Such technical possibilities even lead to the consideration that, in the future, non-interventional studies could be put on an equal footing with randomized, placebo-controlled trials [9].

However, the heterogeneity of EHRs and their diverse archiving requires prior data processing to structure the records and make them analyzable.

Health Level Seven International (HL7) is an organization that defines standards for the exchange, integration, sharing and retrieval of EHRs. It has developed the Fast Healthcare Interoperability Resources (FHIR) standard that facilitates the exchange and analysis of medical data from disparate institutions and electronic data processing systems. [10].

This kind of standardization effort constitutes a necessary step in the development of large data networks that contain patient records from different facilities, thereby enabling large-scale epidemiological analyses. There are initiatives addressing these challenges [11] with considerable recent progress in implementing such data networks [12].

However, before such data networks can be used for epidemiological analyses, it must first be proven that they can be assumed to be representative.

### Objectives

The objective of this study was to compare basic data derived from an AI-enabled data network built and operated by Tiplu GmbH in Germany with data from the German Federal Statistical Office (DESTATIS) to determine whether the dataset from the network can be considered representative and whether epidemiologic analyses based on this dataset would be scientifically valid.

## Methods

### Study design

For proof of representativeness the data sources were evaluated retrospectively as an epidemiological secondary analysis within the framework of a distributed analysis of large, structured data sets.

The hypothesis to be assessed was that the data of the AI-network can be considered representative for epidemiological analyses.

### Ethics vote

There is a positive ethics vote from the Ethics Committee of the University of Witten/ Herdecke (No. S-140/2022).

This retrospective study was conducted using fully anonymized data from medical records and administrative databases (DESTATIS and ML data). All data were de-identified before access and analysis. The study was reviewed and approved by the Ethics Committee of the University of Witten/ Herdecke which waived the requirement for informed consent due to the use of anonymized, retrospective data. No written or verbal consent was obtained from participants, as no identifiable information was accessible to the authors at any stage.

### Databases

The German Federal Statistical Office (DESTATIS) provides public and usable access to anonymized data on inpatients in German hospitals through the online database GENESIS and the Federal Health Monitoring System (GBE-Bund).

Tiplu GmbH is a software company specialized in AI-powered solutions in healthcare. It has developed a data network with roughly 10 million interoperable EHRs from different hospitals across Germany. Local servers with access to the hospital information systems and associated subsystems are connected to a central parameter server to enable federated machine learning (ML) and data analyses. Raw EHR data are standardized into interoperable patient records for secondary analyses across the network. In the following this data network and infrastructure for federated ML is referred to as ML-Network. The potential use of the ML-Network for third parties (other organizations for research and development purposes) is currently being elaborated [12].

### Data protection

To ensure data protection within the ML-Network, EHR data originating from the hospital information systems are automatically anonymized on the local servers using a specific algorithm that changes or removes sensitive content from the data records prior to further processing so that it is no longer possible to identify the respective patients or other persons mentioned in the data records without additional tools and excessive efforts. According to the definition outlined in the position paper on anonymization published by the German Federal Data Protection Commissioner in June 2020, this constitutes de facto anonymization [13].

Furthermore, data aggregations within the ML-Network are performed in a federated procedure so that only completely anonymous parameters, such as number of cases or statistics on case characteristics, are transmitted.

### Variables

Both data sets included age, gender, length of hospitalization, codes of the International Statistical Classification of Diseases and Related Health Problems 10th Revision (ICD-10) of the World Health Organization (WHO) and Operation and Procedure Codes (OPS codes), allowing for comparative analysis of these variables.

OPS codes represent the current German adaptation of the International Classification of Procedures in Medicine (ICPM). Initially developed by the WHO in 1978, the ICPM was limited to surgical procedures and subsequently discontinued in 1989. Consequently, numerous countries have devised analogous classifications at the national level, encompassing both surgical and non-surgical procedures.

### General representativeness

The variables age and gender were utilized to illustrate the general epidemiological comparability. The public reference data did not provide age in metric units but did categorize age into distinct age groups. Another variable that was subjected to comparison is the duration of hospitalization. As well, the corresponding data is not available in quantitative form, as groupings were made in the comparative data sets of the German Federal Statistical Office (DESTATIS).

### Medical representativeness

To illustrate the general medical comparability, the 20 most common ICD-10 codes and OPS codes of the respective databases were compared. As a research group from the field of ear, nose, and throat (ENT) medicine, we also conducted a comparison of ENT-specific codes to ensure their comparability. For this purpose, we compared the 10 most common ENT-specific ICD-10 codes and OPS codes from the respective databases.

### Bias and confounder

Socioeconomic position (SEP) [14] has an impact on health and well-being [15], including in Germany [16]. It is therefore plausible that SEP may act as a confounding or biasing variable. In epidemiology, common indicators of socioeconomic position include education and income [17]. However, these data were not available in the analyzed data sets. An alternative dependent variable could have been the place of residence. Due to data protection regulations, however, this information was not accessible. As a result, the analysis was conducted on a regional level. This methodological limitation introduces the risk of systemic bias due to the relatively imprecise nature of the analysis. To account for socioeconomic confounding factors, the ICD-10 codes F10.- to F15.- and F19.- were additionally analyzed. These codes reflect substance use disorders, which are known to correlate with socioeconomic status [18]. Furthermore, obesity serves as an additional indicator of socioeconomic status [19]. Consequently, the ICD-10 codes E66.- were also subjected to comparison.

### Statistical methods

Since the comparative datasets of the German Federal Statistical Office (DESTATIS) provide the original metric data “age” and “length of hospitalization” in groups that are not equally structured, only a qualitative evaluation of the data was possible.

For these non-metric, nominally scaled variables, relative frequencies were calculated and compared descriptively by presenting them alongside the corresponding public reference data in tabular and graphical form. Where appropriate, percentage point differences were reported to illustrate deviations. This approach allowed a direct and transparent assessment of concordance between the study sample and the reference population.

No statistical hypothesis testing (SHT) was performed. Given the very large datasets, even minimal effect sizes would most likely have reached statistical significance without necessarily being of clinical or epidemiological relevance [20,21]. Consequently, we focused on the descriptive presentation of differences. In line with methodological considerations raised in the literature, the assessment of whether observed deviations are meaningful is therefore reserved for the clinical and epidemiological discussion [20,21].

The following factors were defined a priori as clinically and epidemiologically relevant dimensions to be considered when discussing observed deviations:

- Morbidity and risk profiles (ICD-10 and OPS codes and documented socioeconomic risk factors)
- Demographic structure (age and sex)
- Care structures and intensity (e.g., length of hospital stay)
- Regional distribution

These parameters provide the necessary contextual clinical framework to evaluate the potential clinical relevance of descriptive differences between the study population and the reference data. The selection of these dimensions is consistent with established methodological frameworks regarding external validity, data quality, and population comparability in observational and real-world data research [22–24].

The data from DESTATIS and aggregated data from the ML-Network were processed using the programs Excel and R version 4.5.1 (The R Foundation for Statistical Computing).

### Data access and confidentiality

The DESTATIS data were accessed between 04/29/2023 and 12/03/2023.

The ML data were accessed on 05/26/2023 and 10/30/2024.

The authors did not have access to information that could identify individual participants during or after data collection.

## Results

### Data basis

The ML-Network contained EHR data since the year 2016. The comparative, public databases DESTATIS, on the other hand, provided data up to the year 2022 at the time of the evaluation. Consequently, all available data from 2016 to 2022 were included in the analysis.

In 2016, there were 1951 hospitals in Germany. This number fell to 1893 by 2022 [25]. The ML-Network contained data on inpatients from 96 hospitals in Germany at the time of data aggregations for this study in April 2023. It therefore represented 5.0% of the German hospitals.

As mentioned above, for data protection reasons, Germany, with its 84 million inhabitants, was divided into 4 groups. The “North” of Germany, with around 15 million inhabitants, comprised the federal states of Bremen, Hamburg, Mecklenburg-Western Pomerania, Lower Saxony, and Schleswig-Holstein. The “West” referred to data from the federal states of North Rhine-Westphalia, Hesse, Rhineland-Palatinate, and Saarland, and thus referred to around 29.3 million inhabitants. 15 million inhabitants came from the “East” of Germany with the federal states of Brandenburg, Berlin, Saxony, Saxony-Anhalt, and Thuringia. The “South”, with a population of 24.1 million, included the federal states of Bavaria and Baden-Wuerttemberg.

**Fig 1:**
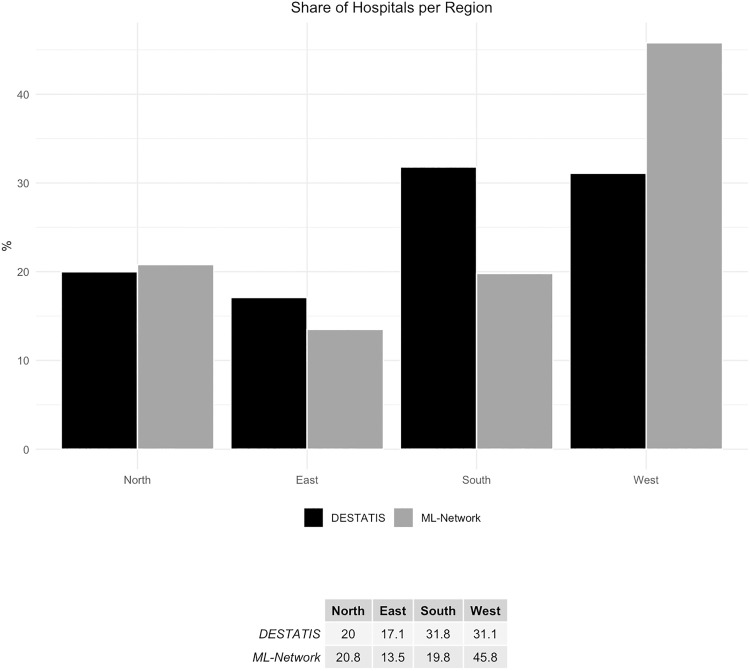
Share of hospitals per region.

It is noteworthy that the hospitals from which the data for both reference databases originate are distributed differently across these country groups (Fig 1).

**Fig 2:**
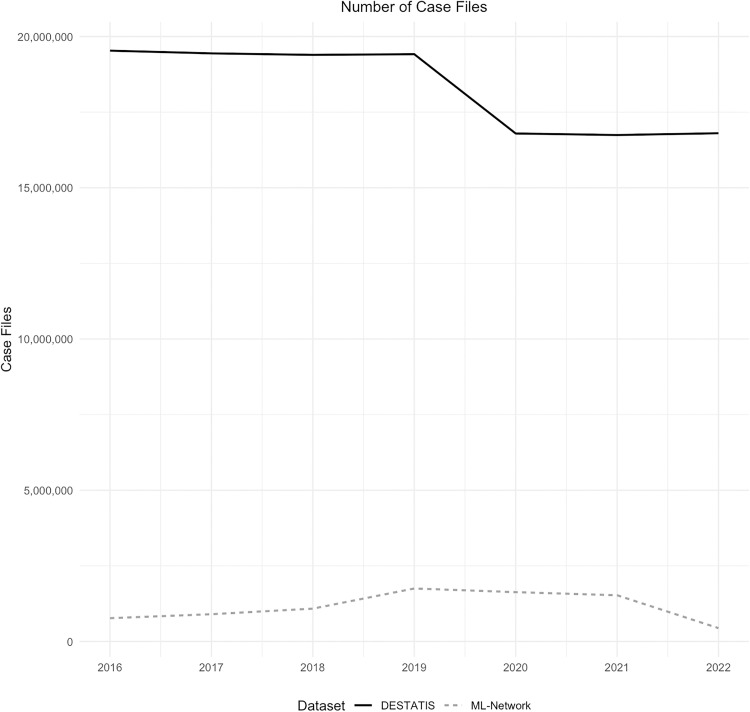
Number of case files.

EHR data sets of 8,106,105 cases from the ML-Network were analyzed, showing a peak for the years 2019–2021. By contrast, the case numbers in the reference data showed a decline since 2019. Overall, the data from the ML-Network represents 6.3% of the comparative data set (Fig 2).

**Fig 3:**
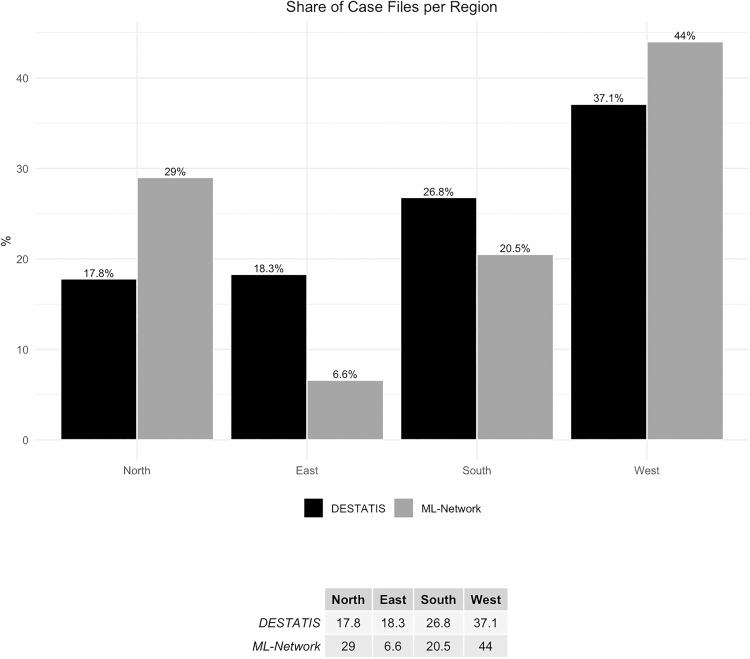
Share of case files per region.

The cooperating clinics and therefore the analyzed cases of the ML-network were not distributed evenly across the Federal State groups (Fig 3).

### General representativeness

#### Age distribution.

**Fig 4:**
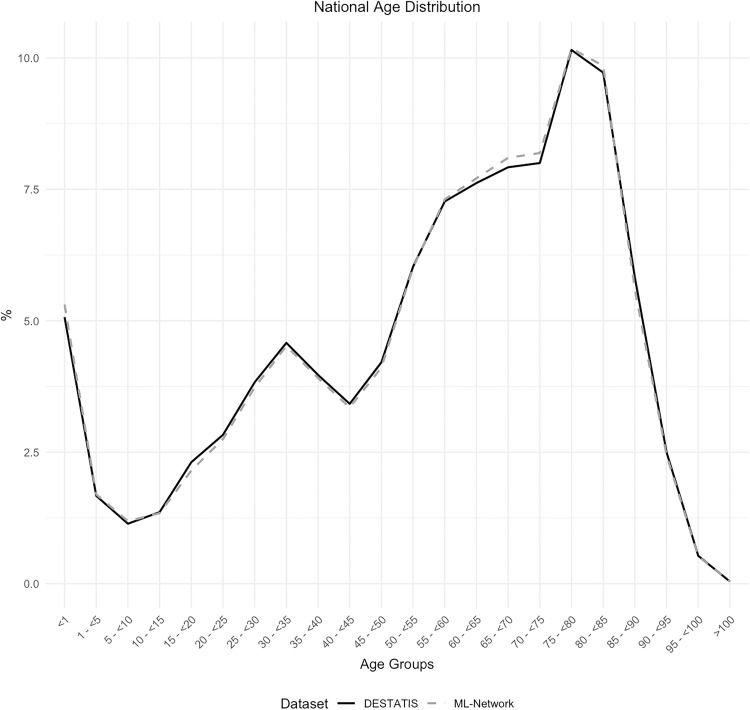
National age distribution.

**Fig 5:**
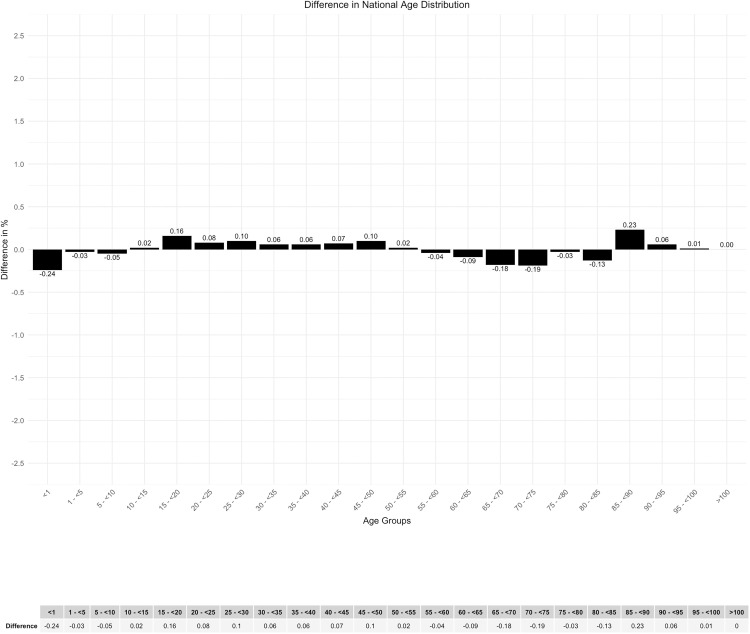
Difference in national age distribution.

The graphical representation of the age distribution by age groups demonstrated a high degree of overlap between the curves of the two data sets utilized for comparison (Fig 4). The analysis of the difference in age distribution between the two data sets revealed a maximum discrepancy of 0.24% (Fig 5).

**Fig 6:**
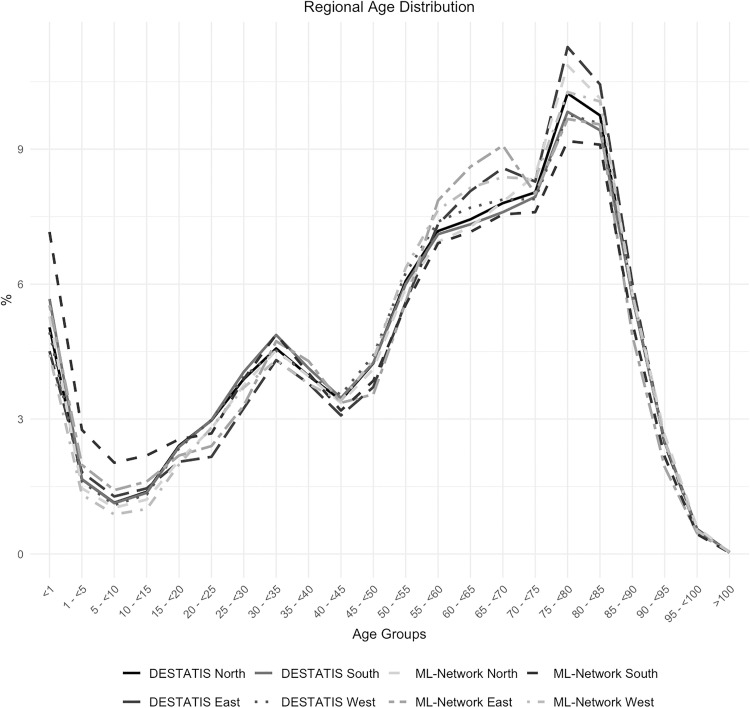
Regional age distribution.

**Fig 7:**
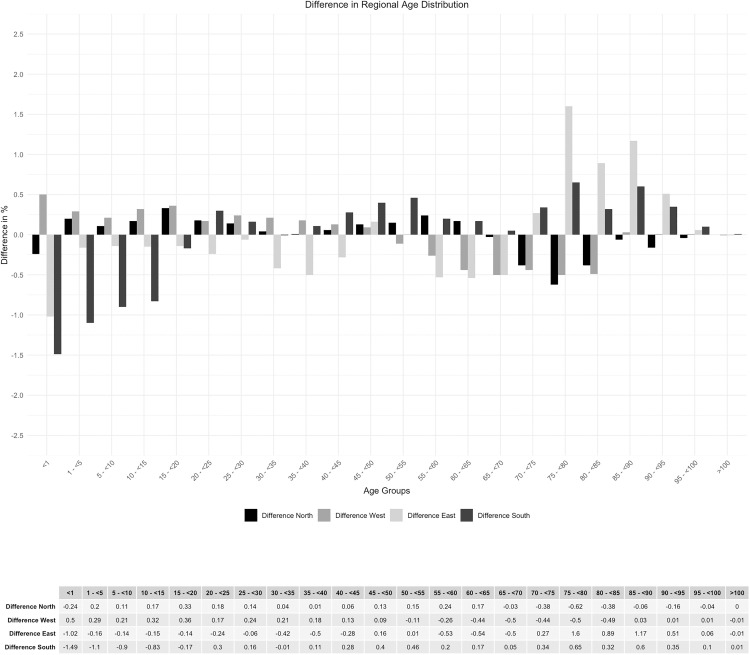
Difference in regional age distribution.

A sub-analysis of the age group distribution by region shows a maximum deviation of 1.6%, which can be considered minor (Figs 6 and 7).

#### Gender distribution.

**Fig 8:**
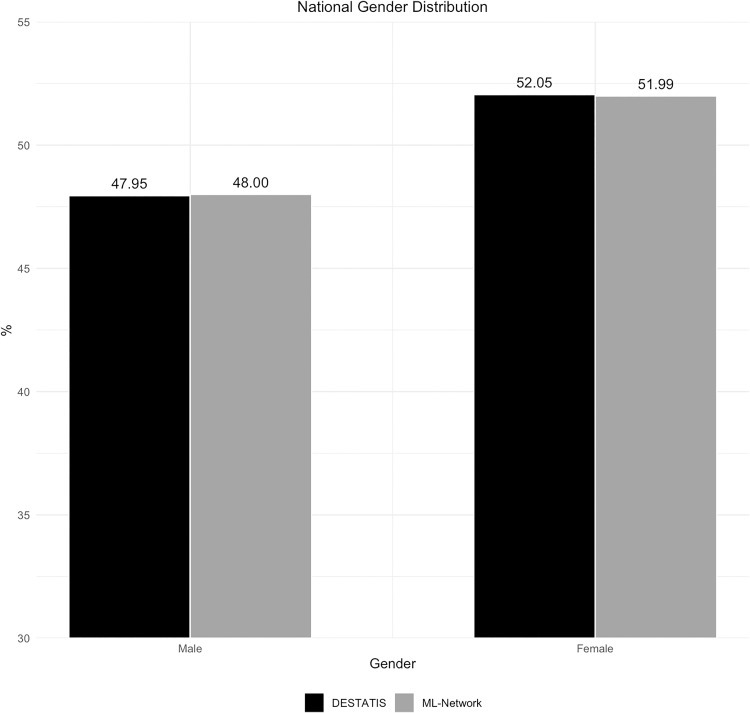
National gender distribution.

**Fig 9:**
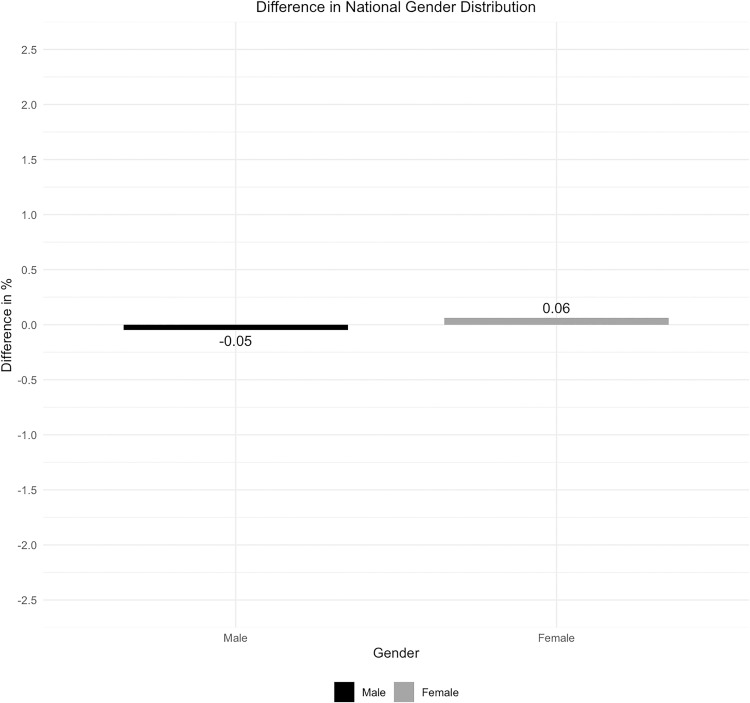
Difference in national gender distribution.

The analysis of gender distribution also showed good congruence between the two compared datasets (Fig 8). The proportion of individuals with an undefined gender in the DESTATIS dataset was found to be 0%, while in the ML dataset it was 0.01%. Due to this low occurrence, these data were not included in the graphs. The differences between the compared data sources regarding the gender distribution were consistently small (Fig 9).

**Fig 10:**
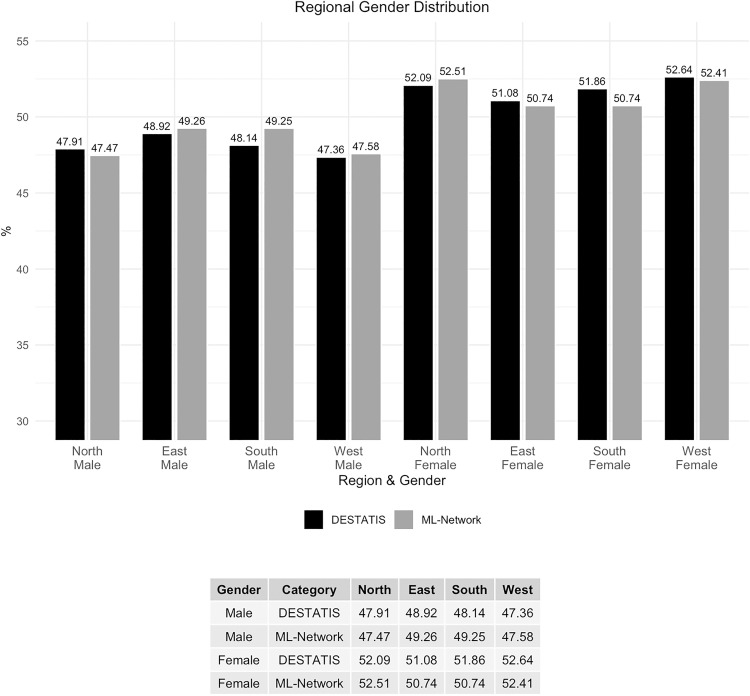
Regional gender distribution.

**Fig 11:**
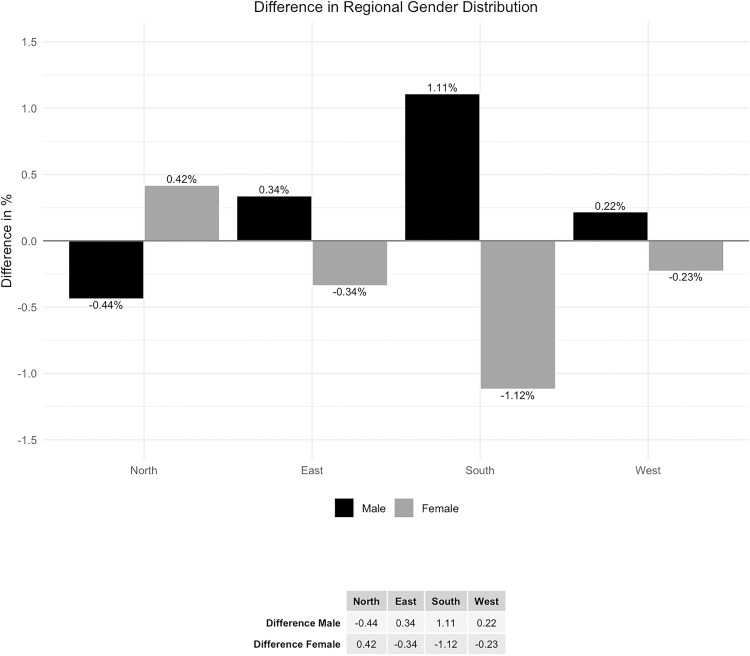
Difference in regional gender distribution.

The gender distribution analyzed by region shows a maximal deviation of 1.12% (Figs 10 and 11).

#### Length of hospitalization.

**Fig 12:**
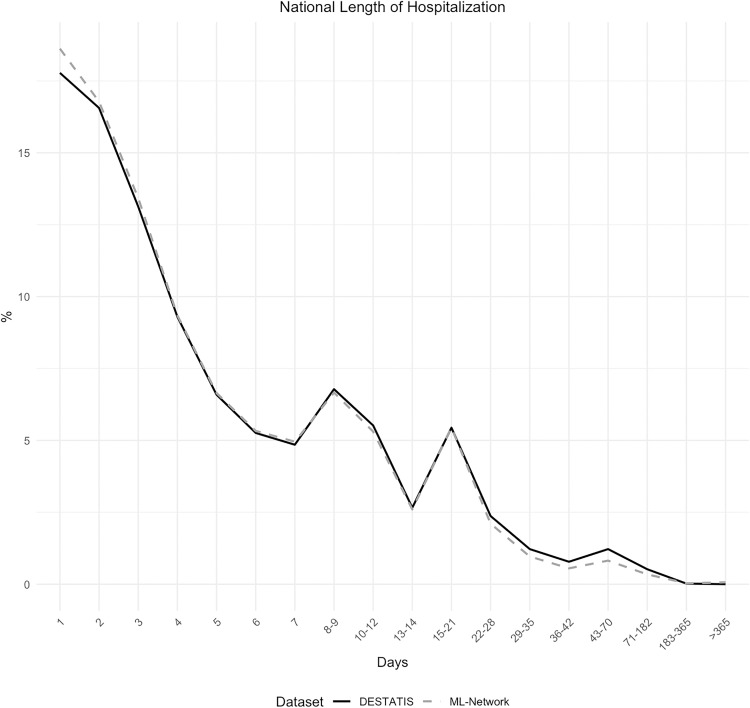
National length of hospitalization.

**Fig 13:**
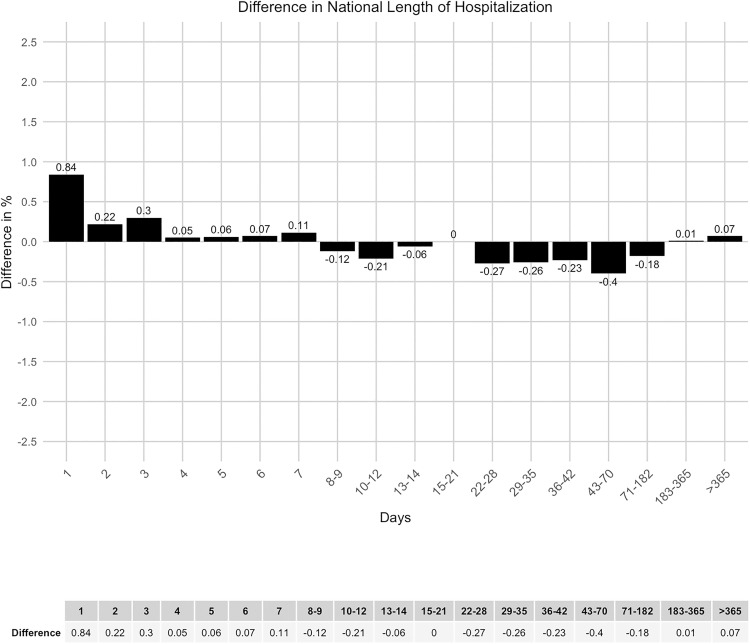
Difference in national length of hospitalization.

**Fig 14:**
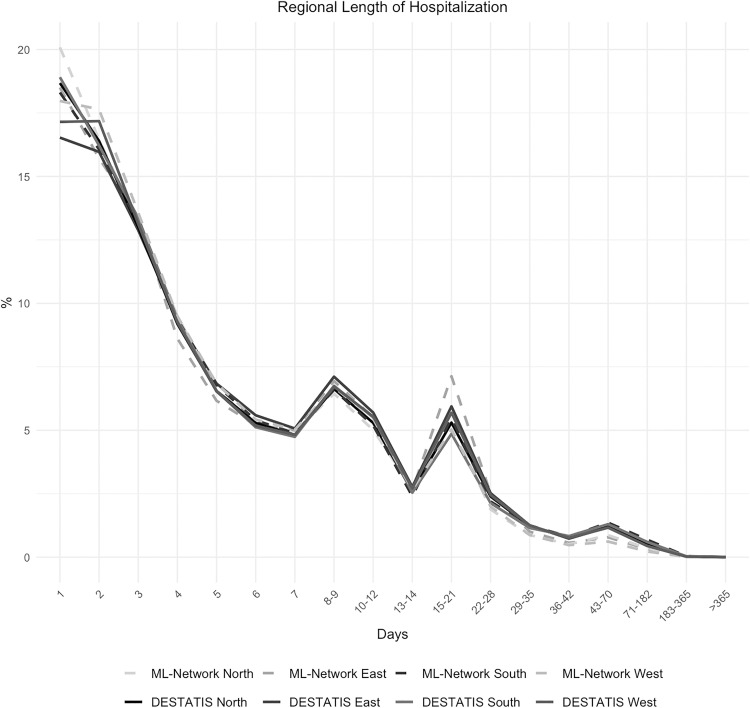
Regional length of hospitalization.

**Fig 15:**
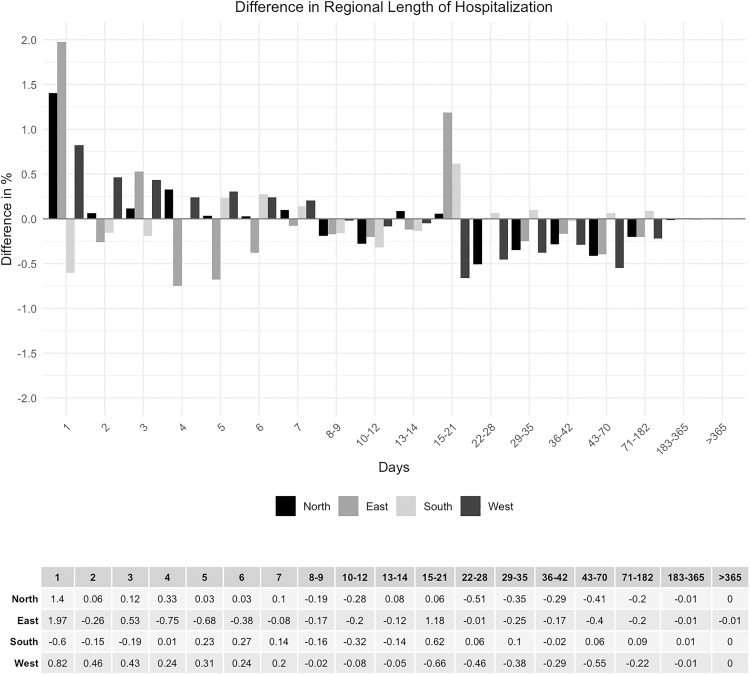
Difference in regional length of hospitalization.

The analysis of the duration of hospitalization demonstrated strong concordance between national and regional data, with a maximum discrepancy of 0.84% (Figs 12 and 13). The same applies to the regional sub-analysis (Figs 14 and 15).

### Medical representativeness

To verify medical representativeness, the most common ICD-10 codes and OPS codes of each database were selected. The results for each database were first ranked separately and then compared.

The primary aim was to compare how many of the codes found in DESTATIS were also found in the ML-Network among the most frequent codes.

Secondly, the percentage frequency of the codes found was compared and thirdly, the ranking shift in the comparison of the databases was analyzed.

#### General medical representativeness.

Of the 20 most frequent ICD-10 codes in the DESTATIS database, 19 were also among the 20 most frequent in the ML-Network. The code K40 “Hernia inguinalis” ranked 20th (0.94%) in DESTATIS data was ranked 22nd (0.73%) in the ML-Network data. The code C50 “Malignant neoplasm of breast” was ranked 20th in the ML-Network dataset (0.84%), while it was ranked 31st in the DESTATIS dataset (0.74%). Therefore, 21 ICD-10 codes were used to compare frequencies (Table 1).

**Table 1:** Most frequent ICD-10 codes in the DESTATIS- and ML- Databases.

ICD-10 code	Disease
Z38	Liveborn infants according to place of birth
I50	Congestive heart failure
I48	Atrial fibrillation and flutter
I63	Cerebral infarction
S06	Intracranial injury
C34	Malignant neoplasm of bronchus and lung
J44	Other chronic obstructive pulmonary disease
I25	Chronic ischemic heart disease
K80	Cholelithiasis
I21	Acute myocardial infarction
M16	Coxarthrosis
I70	Atherosclerosis
F10	Mental and behavioral disorders due to use of alcohol
I20	Angina pectoris
S72	Fracture of femur
M17	Gonarthrosis
I10	Essential (primary) hypertension
J18	Pneumonia, organism unspecified
M54	Back pain
C50	Malignant neoplasm of breast
K40	Inguinal hernia

**Fig 16:**
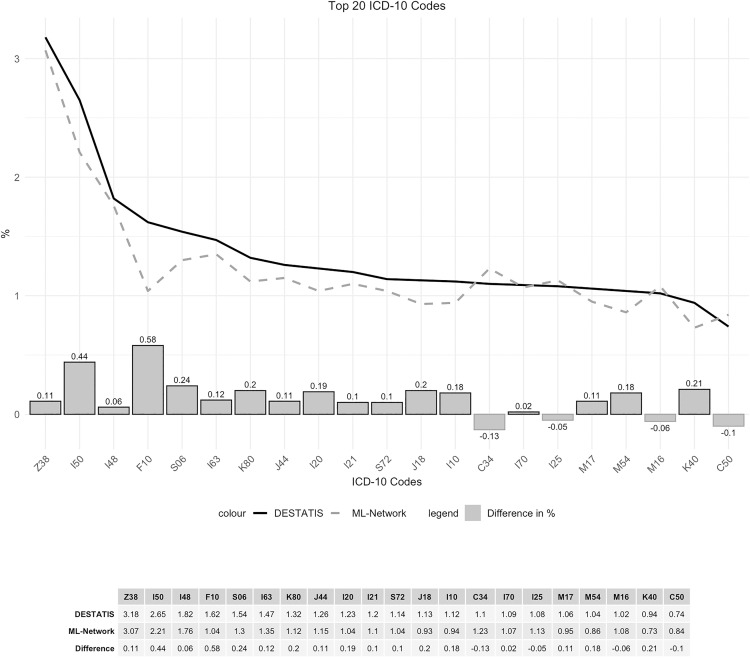
Top 20 ICD-10 codes.

**Fig 17:**
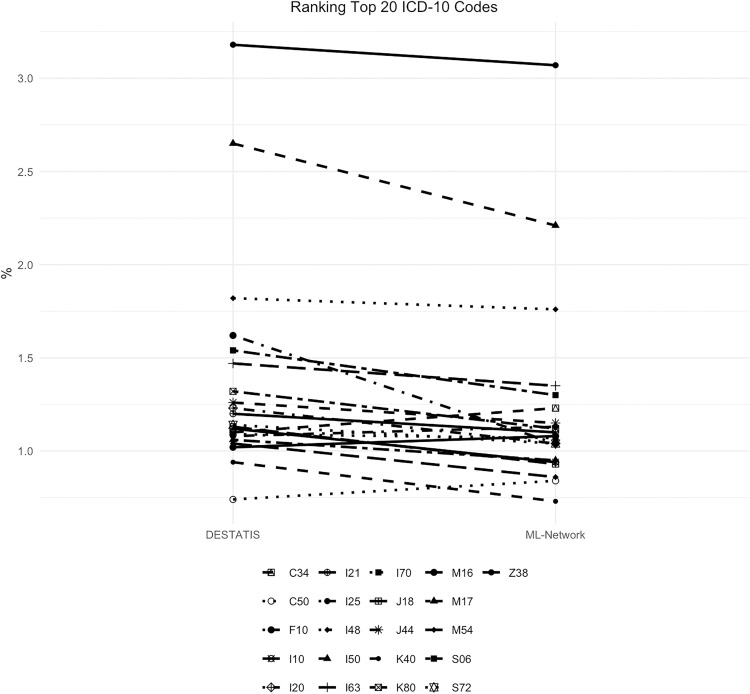
Ranking top 20 ICD-10 codes.

The frequency curves demonstrate a high degree of congruence, with a maximum difference of 0.58% for the code F10 (Fig 16). Although the ranking analysis indicated some shifts in ranking, the gaps between the ranks are so narrow that even minor differences in frequency result in shifts in ranking (Fig 17).

A review of the 20 most frequently occurring OPS codes in the DESTATIS database revealed that 18 of these were also among the 20 most frequent codes in the ML-Network. OPS code 8–837 “Percutaneous transluminal vascular intervention on the heart and coronary vessels”, which ranked 13th (1.13%) among the top 20 OPS codes in the DESTATIS dataset, ranked 29th (0.60%) in the ML-Network dataset. Furthermore, the code 1-207 “Electroencephalography [EEG]” ranked 19th (0.88%) in the DESTATIS dataset, but 22nd (0.83%) in the ML-Network dataset. Regarding the ML-Network data, OPS codes 5–984 “Microsurgical technique” and 8-98f “Complex intensive care treatment (basic procedure)” were among the 20 most frequently coded procedures, holding position 16 (0.94%) and 20 (0.86%). These codes held the 23rd (0.72%) and 22nd (0.77%) position in the DESTATIS dataset. Accordingly, 22 OPS codes were included in the subsequent analysis (Table 2).

**Table 2:** Most frequent OPS codes in the DESTATIS- and ML- Databases.

OPS code	Procedure in German	Procedure in English
9-984	Pflegebedürftigkeit	Need for care
8-930	Monitoring von Atmung, Herz und Kreislauf ohne Messung des Pulmonalarteriendruckes und des zentralen Venendruckes	Monitoring of respiration, heart, and circulation
3-200	Computertomographie des Schädels mit Kontrastmittel	Computed tomography of the skull with contrast agent
3-990	Computergestützte Bilddatenanalyse mit 3D-Auswertung	Computer-aided image data analysis with 3D evaluation
1-632	Diagnostische Ösophagogastroduodenoskopie	Diagnostic esophagogastroduodenoscopy
3-225	Computertomographie des Abdomens mit Kontrastmittel	Computed tomography of the abdomen with contrast agent
8-83b	Zusatzinformationen zu Materialien	Additional information on materials
3-222	Computertomographie des Thorax mit Kontrastmittel	Computed tomography of the thorax with contrast agent
8-800	Transfusion von Vollblut, Erythrozytenkonzentrat und Thrombozytenkonzentrat	Transfusion of whole blood, erythrocyte concentrate, and thrombocyte concentrate
1-275	Transarterielle Linksherz-Katheteruntersuchung	Transarterial left heart catheterization
1-208	Registrierung evozierter Potenziale	Registration of evoked potentials
9-262	Postnatale Versorgung des Neugeborenen	Postnatal care of the newborn
8-837	Perkutan-transluminale Gefäßintervention an Herz und Koronargefäßen	Percutaneous transluminal vascular intervention on the heart and coronary vessels
1-440	Endoskopische Biopsie an oberem Verdauungstrakt, Gallengängen und Pankreas	Endoscopic biopsy of the upper digestive tract, bile ducts and pancreas
1-650	Diagnostische Koloskopie	Diagnostic colonoscopy
8-831	Legen und Wechsel eines Katheters in zentralvenöse Gefäße	Placing and changing a catheter in central venous vessels
3-800	Native Magnetresonanztomographie des Schädels	Native magnetic resonance imaging of the skull
1-710	Ganzkörperplethysmographie	Whole-body plethysmography
1-207	Elektroenzephalographie [EEG]	Electroencephalography [EEG]
9-401	Psychosoziale Interventionen	Psychosocial interventions
8-98f	Aufwendige intensivmedizinische Komplexbehandlung (Basisprozedur)	Complex intensive care treatment (basic procedure)
5-984	Mikrochirurgische Technik	Microsurgical technique

**Fig 18:**
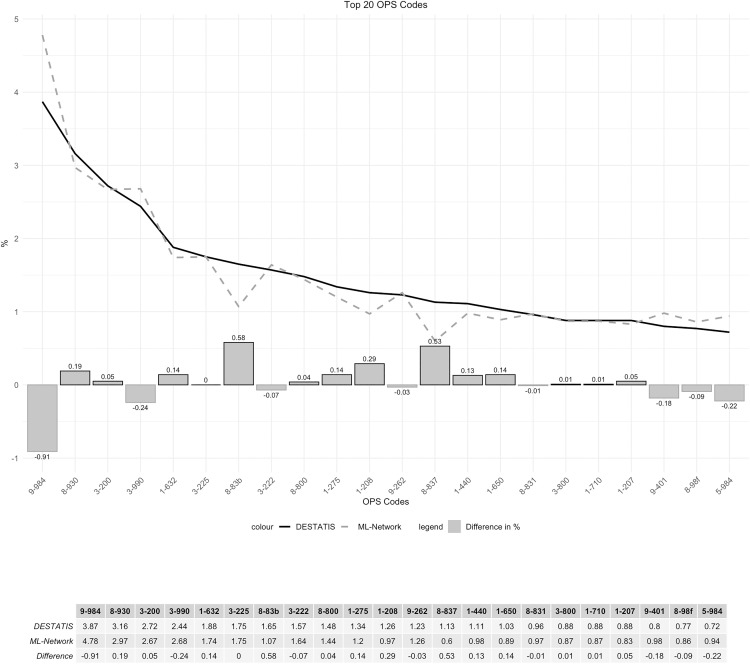
Top 20 OPS codes.

**Fig 19:**
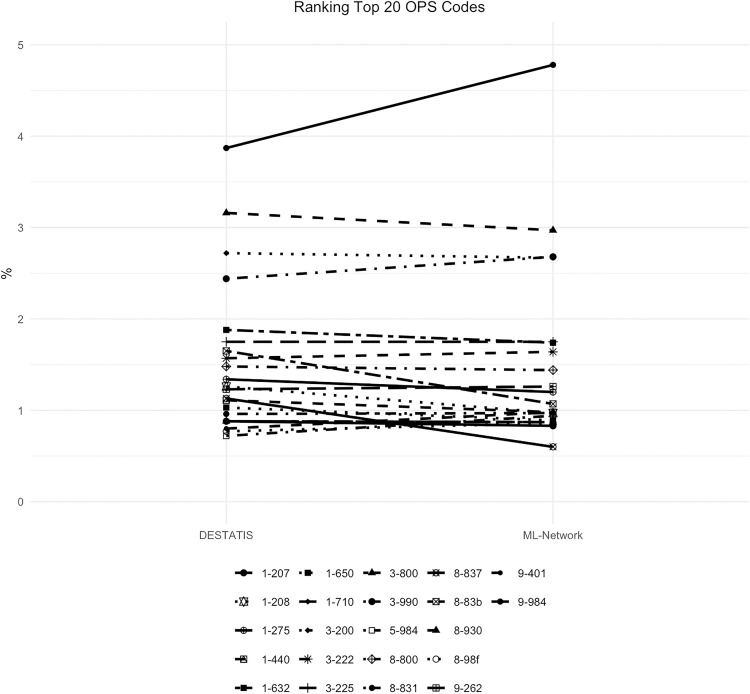
Ranking top 20 OPS codes.

The frequency curves were essentially congruent, with a maximum difference of 0.9% (Fig 18). The ranking analysis showed a somewhat wider spread of results for the ML-Network, but without any substantial shifts (Fig 19).

#### ENT-specific representativeness.

9 of the 10 most frequent DESTATIS ENT-specific ICD-10 codes were also found in the ML database among the 10 most frequent codes. 11 codes were therefore included in the subsequent analysis (Table 3).

**Table 3:** Most frequent ENT-specific ICD-10 codes in the DESTATIS- and ML- Databases.

ICD-10 code	Disease
J34	Other disorders of nose and nasal sinuses
J35	Chronic diseases of tonsils and adenoids
J32	Chronic sinusitis
H81	Disorders of vestibular function
J38	Diseases of vocal cords and larynx, not elsewhere classified
R04	Hemorrhage from respiratory passages
J36	Peritonsillar abscess
H91	Other hearing loss
C32	Malignant neoplasm of larynx
G47	Sleep disorders
H90	Conductive and sensorineural hearing loss

**Fig 20:**
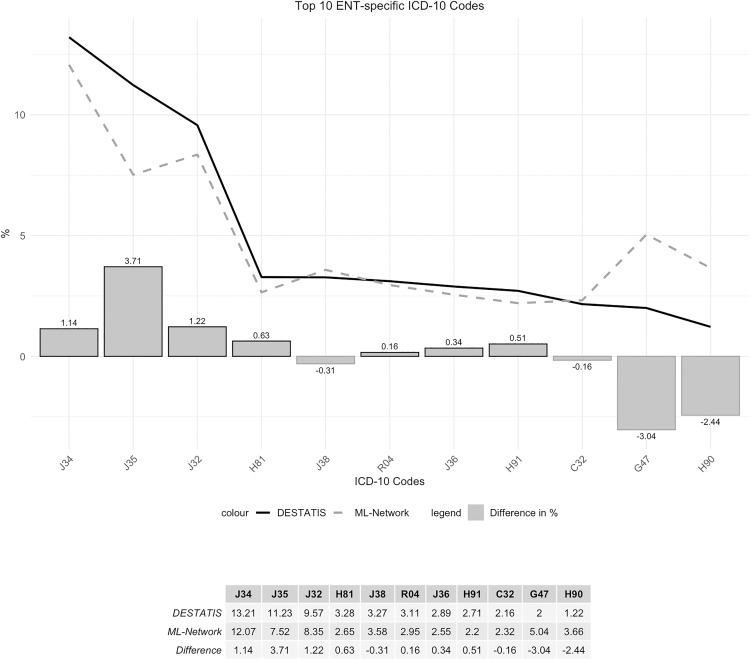
Top 10 ENT-specific ICD-10 codes.

**Fig 21:**
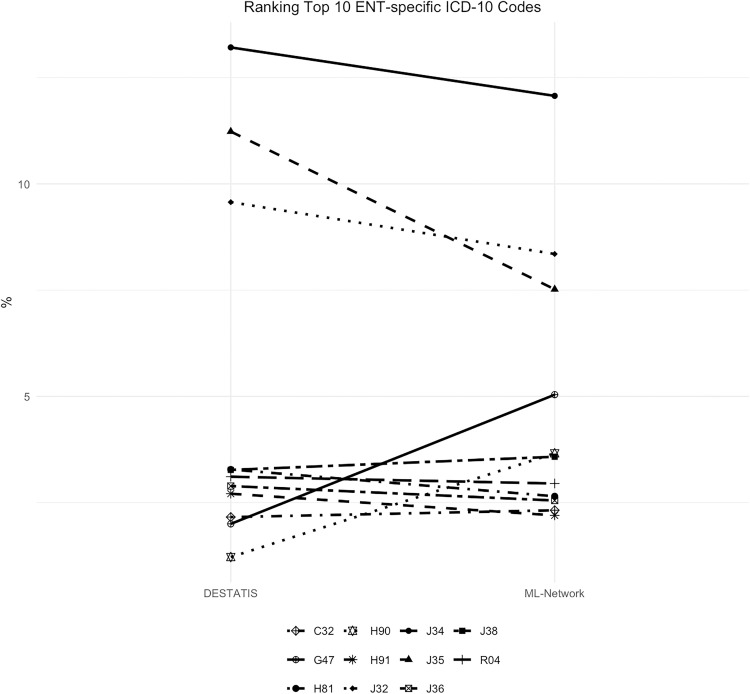
Ranking Top 10 ENT-specific ICD-10 codes.

3 of the codes examined showed noticeable differences in frequency, which can be seen in the comparison of the curve profile. The maximum deviation was 3.71%. (Fig 20). These differences in frequency lead to corresponding shifts in ranking (Fig 21).

It is noteworthy, that some of the divergent codes refer to the same underlying condition but differ in their level of specificity. For example, the top 10 ENT-specific ICD-10 codes in the DESTATIS dataset include H91 “Other hearing loss” (2.71% DESTATIS vs 2.2% ML-Network), while the top 10 ENT-specific ICD-10 codes in the ML-Network dataset include H90 “Conductive and sensorineural hearing loss” (3.66% ML-Network vs 1.22% DESTATIS). With regard to further notable shifts in ranking, it is evident that the ICD-10 code G47 “Sleep disorders” was coded more frequently in the ML-Network dataset (5.04%) than in the DESTATIS dataset (2.0%). As a result, it ranks fourth among the Top 10 ENT-specific ICD-10 codes in the ML-Network data, whereas it ranks tenth in the DESTATIS data. However, since it still appears among the top 10 codes under investigation, the difference is negligible from a clinical perspective. Regarding the code J35 “Chronic diseases of tonsils and adenoids”, a notable difference in percentage distribution can be observed, resulting in a shift in the ranking. In the DESTATIS dataset, the code ranks second (11.23%), while in the ML-Network dataset it ranks third (7.52%), despite the noticeable deviation in frequency.

For the ENT-specific OPS codes, again, 9 of the 10 most frequent DESTATIS codes were among the 10 most frequent ML-Network codes. Therefore, 11 codes were included in the analysis (Table 4).

**Table 4:** Most frequent ENT-specific OPS codes in the DESTATIS- and ML- Databases.

OPS code	Procedure in German	Procedure in English
5-215	Operationen an der unteren Nasenmuschel [Concha nasalis]	Operations on the inferior turbinate [concha nasalis]
5-214	Submuköse Resektion und plastische Rekonstruktion des Nasenseptums	Submucosal resection and plastic reconstruction of the nasal septum
5-281	Tonsillektomie (ohne Adenotomie)	Tonsillectomy (without adenectomy)
5-224	Operationen an mehreren Nasennebenhöhlen	Surgery on multiple paranasal sinuses
5-200	Parazentese [Myringotomie]	Paracentesis [myringotomy]
5-311	Temporäre Tracheostomie	Temporary tracheostomy
5-285	Adenotomie (ohne Tonsillektomie)	Adenectomy (without tonsillectomy)
5-222	Operation am Siebbein und an der Keilbeinhöhle	Surgery on the ethmoid bone and sphenoid sinus
5-221	Operationen an der Kieferhöhle	Surgery on the maxillary sinus
5-300	Exzision und Destruktion von erkranktem Gewebe des Larynx	Excision and destruction of diseased tissue of the larynx
5-195	Tympanoplastik (Verschluss einer Trommelfellperforation und Rekonstruktion der Gehörknöchelchen)	Tympanoplasty (closure of an eardrum perforation and reconstruction of the auditory ossicles)

**Fig 22:**
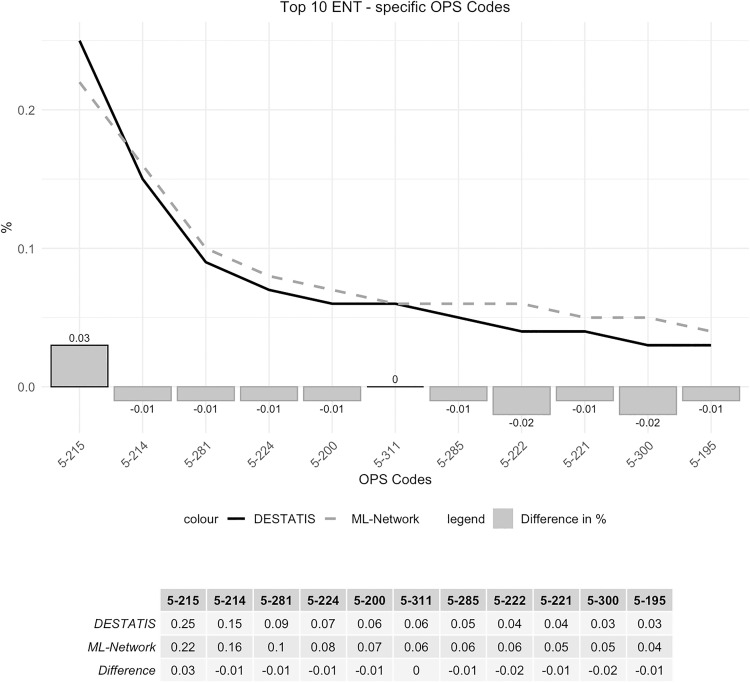
Top 10 ENT-specific OPS codes.

**Fig 23:**
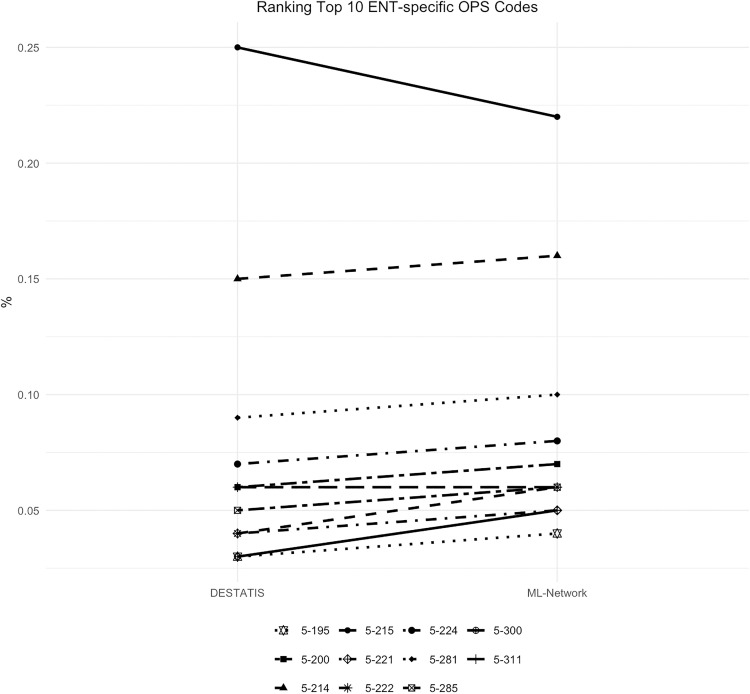
Ranking Top 10 ENT-specific OPS codes.

The frequency analysis of these codes showed good curve congruence with a maximum deviation of 0.03% (Fig 22). Ranking analysis shows only minimal ranking shifts (Fig 23).

The OPS code 5–300 “Excision and destruction of diseased tissue of the larynx“ranks tenth in the ML-Network dataset (0.05%), while it ranks eleventh in the DESTATIS dataset (0.03%). In contrast, the OPS code 5-195 “Tympanoplasty (closure of an eardrum perforation and reconstruction of the auditory ossicles)” holds the tenth position in the DESTATIS dataset (0.04%) but ranks eleventh in the ML-Network dataset (0.04%).

### Socioeconomic confounders

In addition to the regional analyses to control for socioeconomic confounders described above, the distribution of alternative socioeconomic indicators, specifically psychotropic substance abuse and obesity, was examined (Table 5).

**Table 5:** Most frequent socioeconomic confounder associated ICD-10 codes in the DESTATIS- and ML- Databases.

ICD-10 code	ICD-10 Text	Abbreviation
E66	Obesity	Obesity
F10	Mental and behavioural disorders due to use of alcohol	Alcohol
F11	Mental and behavioural disorders due to use of opioids	Opioids
F12	Mental and behavioural disorders due to use of cannabinoids	Cannabinoids
F13	Mental and behavioural disorders due to use of sedatives or hypnotics	Hypnotics
F14	Mental and behavioural disorders due to use of cocaine	Cocaine
F15	Mental and behavioural disorders due to use of other stimulants, including caffeine	Stimulants
F19	Mental and behavioural disorders due to multiple drug use and use of other psychoactive substances	Multiple Drug Abuse

**Fig 24:**
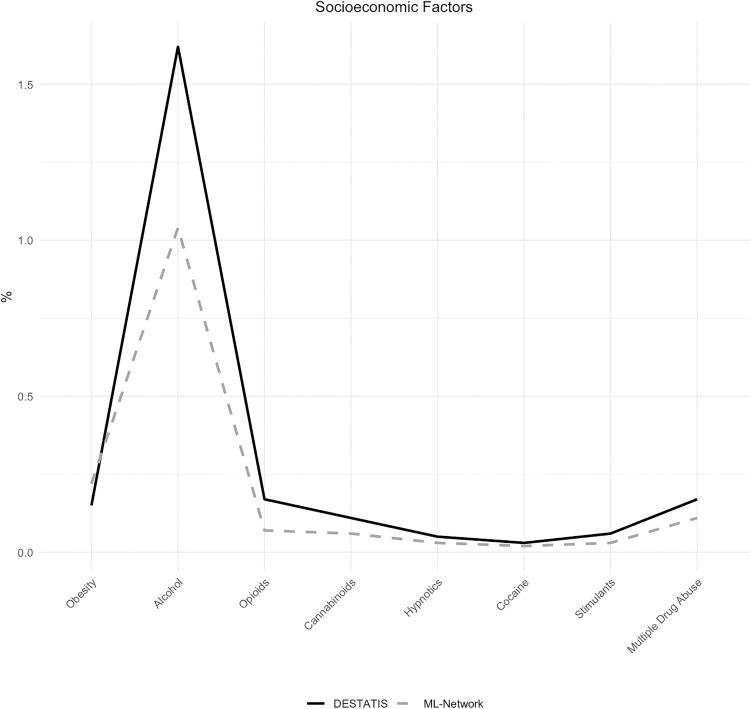
Socioeconomic factors.

**Fig 25:**
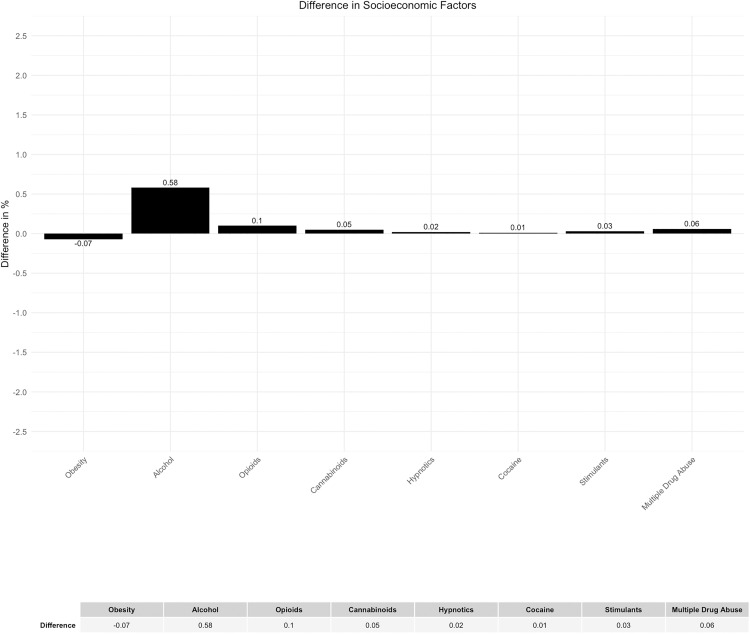
Difference in socioeconomic factors.

A visually noticeable difference was found in the documentation of ICD-10 code occurrences for group F10, which is associated with alcohol-related diseases (Fig 24). However, the discrepancy can be considered minor due to a deviation of 0.58% (Fig 25).

## Discussion

### Key results

The use of retrospective real-world data in AI-supported analyses of large datasets has been discussed extensively in recent years [23]. In this study, we compared publicly available population data from the German Federal Statistical Office (DESTATIS) with data from an AI-enabled hospital data network (ML-Network) provided by Tiplu GmbH. The aim of this comparison was to assess the extent to which the ML-Network reflects the demographic and medical characteristics of the German population.

As predefined in the Methods, the evaluation of representativeness focused on four clinically and epidemiologically relevant dimensions: (1) morbidity and risk profiles, (2) demographic structure, (3) care structures and intensity, and (4) regional distribution. These dimensions were used as the framework for interpreting the descriptive deviations between the ML-Network and the reference data.

Morbidity and risk profiles and therefor medical representativeness was assessed by comparing the most frequent ICD-10 and OPS codes. The most common diagnoses and procedures showed only small absolute differences. Although minor percentage deviations occasionally altered rank order, these shifts did not reflect substantive differences in underlying frequencies. This applied similarly to ENT-specific ICD-10 codes, where no clinically meaningful deviations were observed. Overall, the morbidity and procedure profiles were highly similar across datasets.

Socioeconomic characteristics, included as part of the morbidity and risk profile dimension, showed comparable distributions in both datasets, including origin and documented cofactors such as addiction and obesity. These findings provide no indication of systematic socioeconomic confounding.

Demographic characteristics, particularly age and sex, showed a high level of agreement between the ML-Network and DESTATIS. Because demographics substantially influence morbidity patterns and healthcare utilization, this concordance suggests that the demographic structure of the ML-Network is broadly comparable to that of the general population.

Care structures and intensity were examined using length of hospital stay. No major deviations emerged that would indicate structural differences in care processes between the datasets.

Finally, regional distribution was assessed to identify potential regional biases that might limit generalizability. No evidence of substantial regional distortions was observed.

Taken together, across all four predefined dimensions—demographics, morbidity and risk profiles, care structures, and regional distribution—the ML-Network showed a high degree of alignment with national reference data. Within the constraints of retrospective real-world data, the ML-Network appears suitable for epidemiological and health services research.

### Limitations

A limiting factor that must always be considered in the interpretation of future epidemiological analyses is the fact that the data of the ML-Network examined here is not an archived data set, as is the case with the comparative data from the Federal Statistical Office.

Due to the fluctuation of hospitals feeding data into the ML-Network, the data available at the time of analysis will change accordingly.

The limitations of the presented work include the limited geographical accuracy of the data analysis. A sub-analysis by zip code, for example, would be a valuable addition. Nevertheless, the requisite degree of accuracy could not be generated from the DESTATIS comparative data, nor was it feasible for the ML-Network due to data protection considerations. However, the analysis by region and the integration with socioeconomic co-factors employed in this study can be regarded as a satisfactory substitute.

### Interpretation

The results of the comparison of the databases of the German Federal Statistical Office (DESTATIS) and the AI-enabled data network of Tiplu GmbH (ML-Network) demonstrate a high degree of concordance. Consequently, it can be concluded that epidemiological analyses based on the data of the ML-Network are feasible at both the national and regional levels and justify scientific conclusions.

### Generalizability

The use of large data networks as representative data sources represents a possible advancement in the field of medical research. The technical possibilities offered by artificial intelligence could enable the generation of scientific findings that were previously not possible [26].

To evaluate the advanced possibilities of large data networks, it is crucial to understand the strengths and limitations of the data sources which are currently used for epidemiological research in the German healthcare system.

Clinical registries, for example cancer registries, are frequently used to monitor disease patterns, evaluate treatment outcomes, and inform public health strategies. The German Centre for Cancer Registry Data (ZfKD) collects and harmonizes data from all federal state cancer registries, enabling population-wide analyses of cancer incidence, survival, and mortality [27]. The high coverage of these registries allows for robust epidemiological assessments and the identification of trends in the German healthcare system. Despite these advantages, registry data in Germany face certain limitations, as there are delays in data availability due to the complex reporting and validation processes, which can hinder timely analysis [28]. Furthermore, the granularity of clinical information is often limited; for example, data on comorbidities, treatment decisions, or patient-reported outcomes may be not available or inconsistently recorded [29]. It is known that regional differences in the registry infrastructure and data quality can lead to heterogeneity in data completeness and therefore to a limitation regarding the comparability across federal states [29]. Therefore, they often require supplementation with other data sources to ensure a rational understanding of clinical outcomes. Moreover, it must be acknowledged that clinical registries in Germany exist only for a limited number of diseases [30]. In conclusion, while registry data are invaluable for large-scale observational research, the described limitations need to be carefully considered when interpreting results derived from registry-based studies. To address these limitations, considerable efforts are currently being made to improve the quality of the recorded content and to enhance data accessibility for scientific purposes [31]

The German Federal Statistical Office (DESTATIS) provides a wide array of healthcare-related data that play an important role in health services research. Because DESTATIS data are collected systematically at the national level, they offer comprehensive coverage and standardization, making them particularly suitable for macro-level analyses [32]. A major strength of DESTATIS health data lies in its methodological rigor and legal mandate, which ensures consistent data collection across all federal states, providing valuable insights into healthcare utilization patterns [32]. However, DESTATIS data also faces certain limitations. While the breadth of data is substantial, its clinical depth is limited, as information on patient characteristics such as disease severity or socioeconomic background is often unavailable [33]. Furthermore, the datasets are typically aggregated at the institutional or regional level, which can restrict patient-level analyses and therefore limit the ability to adjust for individual confounders [34]. Additionally, due to privacy regulations, access to microdata is tightly controlled and often subject to significant administrative barriers [27].

In comparison to the data sources currently utilized for epidemiological investigations in the German healthcare system, the ML-Network provides several methodological advantages. Its dataset includes all variables recorded in the electronic health records of the respective hospital information systems, enabling analyses beyond billing-relevant factors, such as ICD-10- or OPS codes. Moreover, the ML-Network allows for semantic analyses of electronic health records data, thereby extending the analytical possibilities. In a current study which will be published separately, we utilized ML-Network data to assess the association between established risk factors for head and neck carcinomas and tumor localization, as well as the incidence of complications relative to tumor site. Collectively, these features position the ML-Network as a particularly valuable resource for advanced epidemiological research, offering substantial improvements over existing registry and DESTATIS datasets.

The dataset analyzed in this study is intended for subsequent use in non-commercial research. For this purpose, Tiplu GmbH aims to provide the ML Network on a non-profit basis for non-commercial data analyses [12]. The representativeness established in the present work constitutes a fundamental prerequisite for its meaningful use.
